# Supplementary material for: Consumption of carotenoids not increased by bacterial infection in brown trout embryos (Salmo trutta)
Source: PLoS One. 2018 Jun 13;13(6):e0198834. doi: 10.1371/journal.pone.0198834 (PMC5999266; doi:10.1371/journal.pone.0198834)
Supplement: S1 Table — Data represent technical replicates (two independent runs of the same sample at different times during ultrahigh-performance liquid chromatography–high resolution mass spectrometry). The CV (or relative standard deviation RSD) is defined as the ratio of the standard deviation (SD) to the mean. The first three letters of the sample name identify the dam, the last three numbers identify the sire, “-C-” stands for sham-treated control, and “-PF-”stands for samples exposed to PF. (PDF) [file pone.0198834.s001.pdf]

Sample	Lutein			Astaxanthin			Zeaxanthin		
	Average (nM)	SD (nM)	CV (%)	Average (nM)	SD (nM)	CV (%)	Average (nM)	SD (nM)	CV (%)
ACW-C-106	2.6	0.2	8	23.9	2.8	12	17.4	0.8	4
ACW-PF-106	2.2	0.1	5	23.2	0.8	4	13.9	1.6	12
ACX-C-107	2.4	0.3	11	10.3	1.9	19	10.0	0.5	5
ACX-PF-107	2.9	0.3	9	15.3	0.7	5	11.9	0.9	8
ACY-C-112	1.6	0.2	13	7.2	0.6	9	11.4	1.4	12
ACY-PF-112	1.4	0.1	9	5.6	0.9	15	8.3	0.9	11
ACZ-C-109	2.6	0.2	8	13.4	0.8	6	20.7	2.0	10
ACZ-PF-109	2.1	0.2	12	9.5	1.2	13	14.3	1.8	13
ADA-C-111	1.7	0.1	5	16.1	0.7	4	9.3	0.4	5
ADA-PF-111	1.5	0.1	7	14.5	1.1	8	8.1	0.9	11
ADB-C-113	2.6	0.3	13	9.6	1.0	10	11.7	1.8	15
ADB-PF-113	2.4	0.2	7	8.9	1.1	12	10.9	0.2	2
ADC-C-110	1.5	0.2	12	4.2	0.9	21	6.5	1.2	19
ADC-PF-110	1.3	0.2	12	3.1	0.5	17	4.6	0.8	18
ADD-C-116	1.6	0.0	3	3.1	0.2	6	3.8	0.5	14
ADD-PF-116	1.9	0.1	5	3.4	0.1	2	5.3	0.1	2
ADF-C-116	1.42	0.03	2	4.2	0.5	12	8.7	0.9	10
ADF-PF-116	1.8	0.2	11	5.3	0.1	1	13.9	3.1	22
ADG-C-114	2.0	0.3	14	10.0	1.3	13	3.7	0.3	8
ADG-PF-114	2.0	0.2	9	11.4	0.8	7	4.2	0.7	16
ACP-101-C-1	2.1	0.1	2	2.7	0.5	19	8.5	1.1	14
ACP-101-PF-1	1.8	0.1	4	2.0	0.3	15	6.1	1.2	19
ACV-104-C-1	2.0	0.1	4	1.4	0.1	5	6.6	0.8	12
ACV-104-PF-1	1.9	0.0	1	1.5	0.1	4	7.1	0.2	3
AEK-151-C-1	2.5	0.2	6	0.6	0.0	3	5.7	0.3	6
AEK-151-PF-1	2.6	0.1	3	0.7	0.0	7	6.5	0.8	13
AEM-150-C-1	2.0	0.3	17	0.7	0.2	24	3.6	0.6	16
AEM-150-PF-1	2.2	0.1	4	0.7	0.0	7	3.8	0.5	12
AEN-147-C-1	2.3	0.2	8	0.9	0.1	17	2.3	0.3	11
AEN-147-PF-1	2.8	0.1	5	1.3	0.1	10	3.4	0.3	9
AEP-148-C-1	3.8	0.1	3	1.6	0.1	4	3.7	0.2	5
AEP-148-PF-1	3.9	0.2	6	1.7	0.1	7	3.7	0.3	9
AER-155-C-1	2.51	0.04	2	4.2	0.0	1	11.0	0.3	3
AER-155-PF-1	2.3	0.1	6	3.8	0.7	19	10.1	1.2	12
AES-155-C-1	2.1	0.1	2	5.0	0.3	5	2.6	0.2	9
AES-155-PF-1	2.1	0.1	4	4.8	0.7	15	2.6	0.2	8
AEV-153-C-1	2.0	0.1	4	6.9	0.1	1	2.8	0.2	8
AEV-153-PF-1	2.48	0.02	1	4.0	0.1	2	2.1	0.2	7
ACK-96-C-1	3.1	0.2	6	10.3	0.3	3	10.2	2.3	22
ACK-96-PF-1	3.0	0.1	4	8.7	1.0	12	8.4	0.6	7

Sample	Lutein			Astaxanthin			Zeaxanthin		
	**Average (nM)**	**SD (nM)**	**CV (%)**	**Average (nM)**	**SD (nM)**	**CV (%)**	**Average (nM)**	**SD (nM)**	**CV (%)**
ACM-94-C-1	2.6	0.2	6	11.8	1.1	9	9.1	1.2	14
ACM-94-PF-1	2.6	0.1	5	12.6	1.8	14	5.6	0.4	7
ACN-95-C-1	2.9	0.1	5	16.1	2.0	12	5.1	0.4	8
ACN-95-PF-1	3.0	0.3	8	16.3	3.9	24	5.4	0.8	14
ACO-100-C-1	2.34	0.02	1	2.1	0.1	3	6.8	0.7	10
ACO-100-PF-1	3.5	0.1	2	2.3	0.1	2	9.9	4.5	45
ACS-99-C-1	2.4	0.1	6	11.2	0.2	1	7.5	1.2	16
ACS-99-PF-1	2.5	0.2	7	15.7	1.4	9	8.1	0.6	7
ACU-108-C-1	2.4	0.1	5	9.1	1.6	18	7.7	1.1	14
ACU-108-PF-1	2.4	0.1	6	9.2	0.4	4	6.6	0.7	10
ADH-55-C-1	2.6	0.1	4	6.9	0.9	12	5.3	0.7	14
ADH-55-PF-1	2.9	0.2	8	8.1	0.2	3	5.8	0.9	15
AEQ-156-C-1	2.9	0.2	6	4.5	0.2	4	5.0	0.5	10
AEQ-156-PF-1	2.6	0.2	6	3.5	0.1	3	3.9	0.3	8
AET-152-C-1	3.0	0.1	3	10.2	1.2	11	8.4	1.0	12
AET-152-PF-1	3.1	0.2	5	10.6	1.4	13	8.0	1.0	12
AEU-145-C-1	2.7	0.2	9	8.4	1.6	19	9.5	1.7	17
AEU-145-PF-1	2.9	0.2	7	8.4	0.9	10	9.0	0.7	7
ACJ-97-C-1	3.1	0.7	22	18.2	1.9	10	17.9	3.2	18
ACJ-97-PF-1	2.8	0.8	27	15.9	3.0	19	15.1	3.1	21
ACL-97-C-1	2.4	0.4	17	4.4	0.4	9	18.6	4.2	22
ACL-97-PF-1	2.2	0.1	7	4.4	0.2	6	19.8	2.6	13
ACR-102-C1	3.5	0.6	16	24.3	1.6	7	24.2	4.3	18
ACR-102-PF-1	2.9	0.5	17	21.0	1.1	5	20.9	5.0	24
ACT-105-C-1	3.0	0.5	15	6.6	0.6	9	17.0	3.1	18
ACT-105-PF-1	3.0	0.6	21	6.8	2.0	29	17.8	7.4	41
AEL-149-C-1	2.0	0.2	12	3.1	1.7	56	9.0	1.7	19
AEL-149-PF-1	1.9	0.2	9	2.9	0.1	5	7.0	0.7	10
ADE-115-C-1	3.0	0.3	11	32.5	5.8	18	8.1	1.4	18
ADE-115-PF-1	3.0	0.4	12	36.2	2.8	8	8.5	1.1	13
