# Supplementary figures and images for: Consumption of carotenoids not increased by bacterial infection in brown trout embryos (Salmo trutta)
Source: PLoS One. 2018 Jun 13;13(6):e0198834. doi: 10.1371/journal.pone.0198834 (PMC5999266; doi:10.1371/journal.pone.0198834)

(a) Hatching time

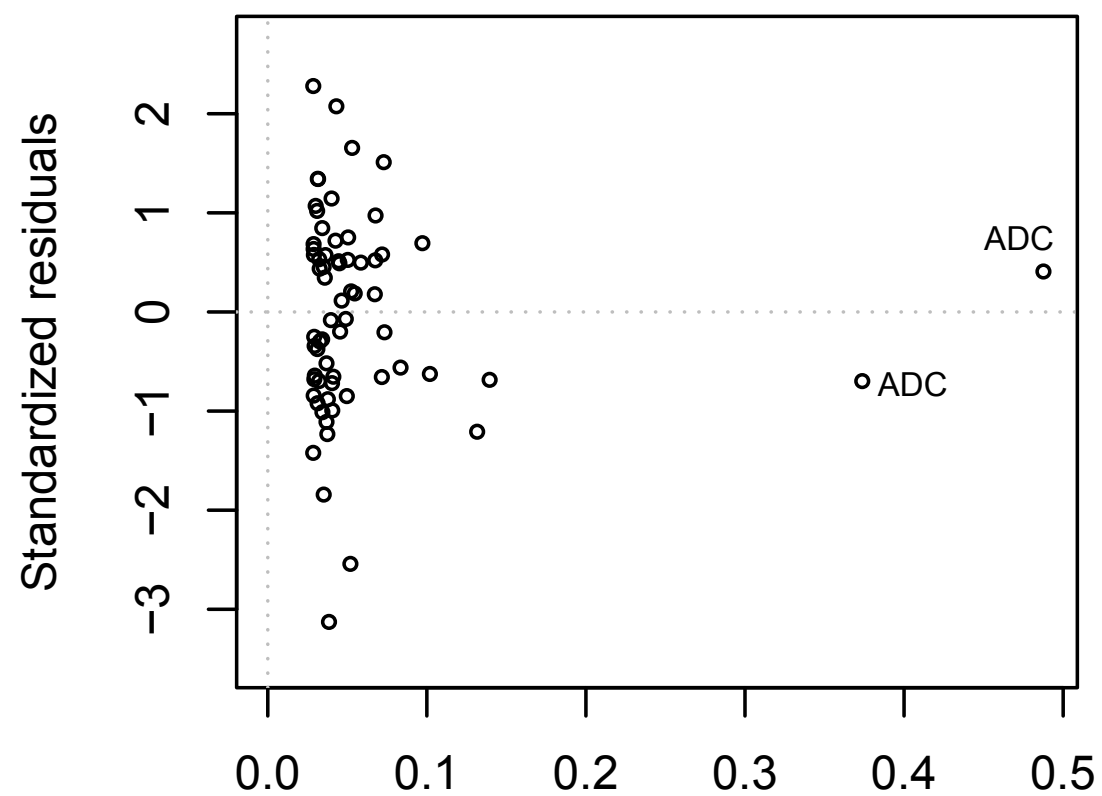

(b) Hatchling length

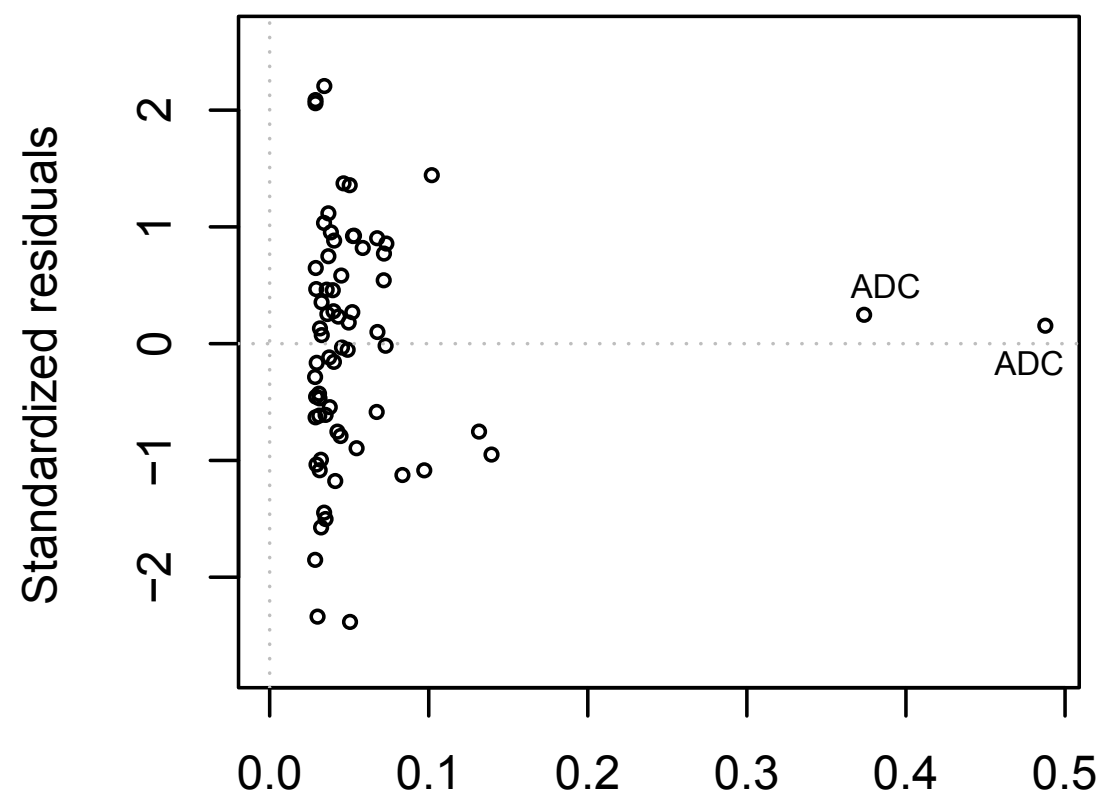

(c) Larval growth

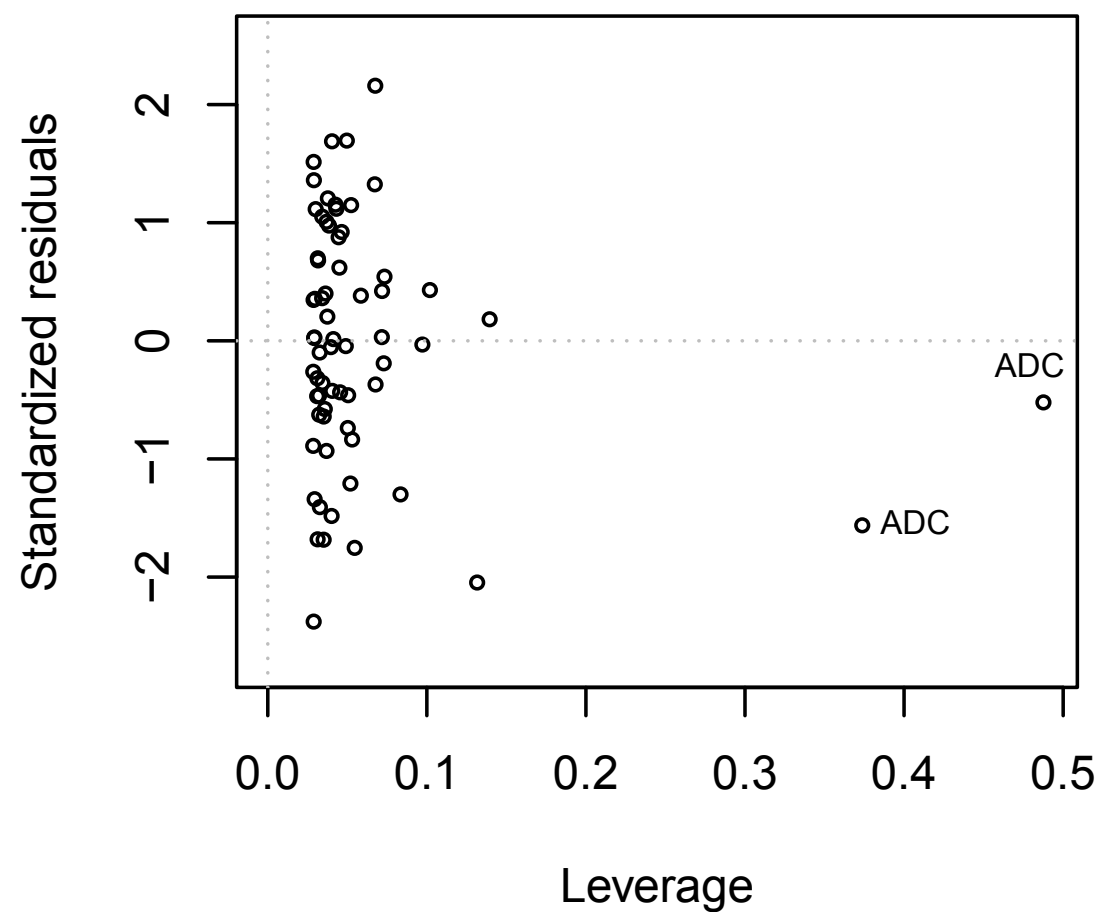

(d) Yolk sac volume at hatching

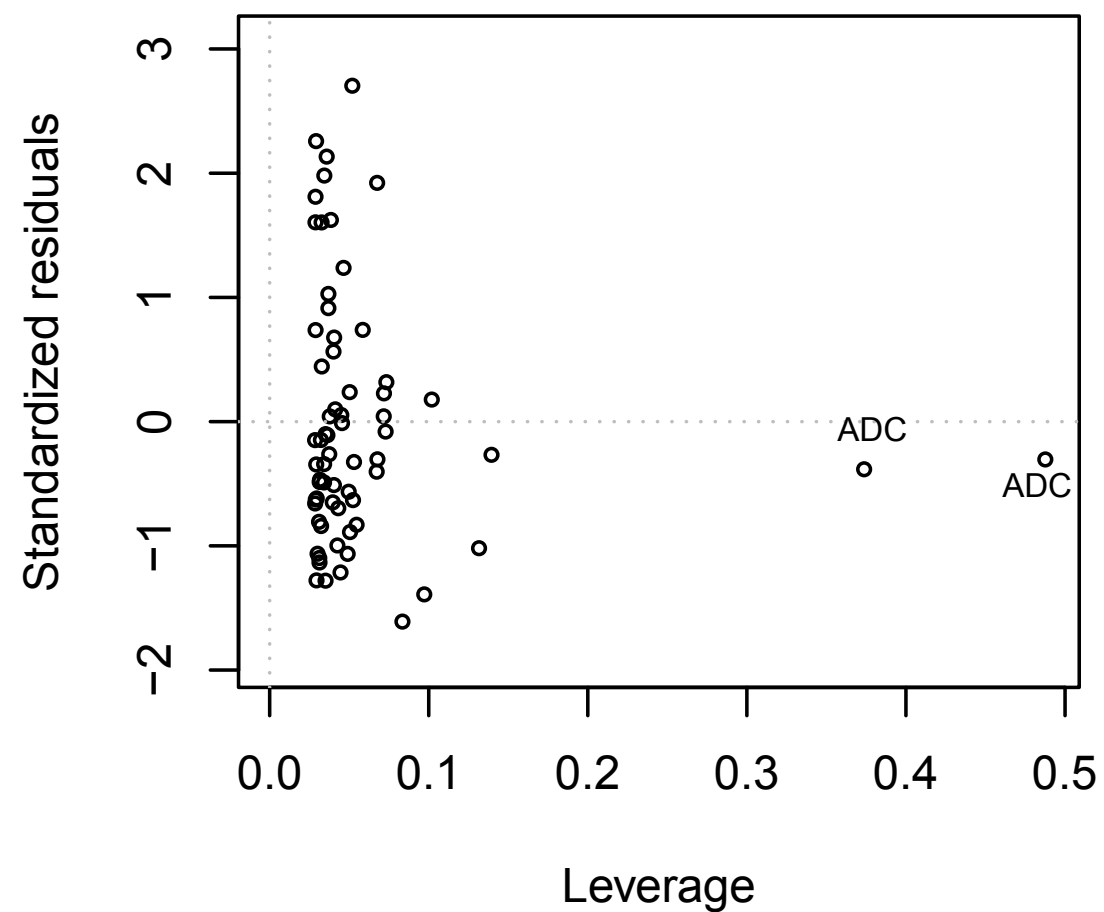

Supplement: S1 Fig — Panels represent data of models on the effects of treatment, change in lutein, and their interaction on (a) hatching time, (b) hatchling length, (c) larval growth, and (d) yolk sac volume at hatching. Each female represents two data points (one for PF exposure and one for sham treatment). The outlier female (“ADC”) is indicated in each panel. This outlier female also presented disproportional statistical leverage in models on embryo traits and change in astaxanthin. (PDF) [file pone.0198834.s002.pdf]

(a)

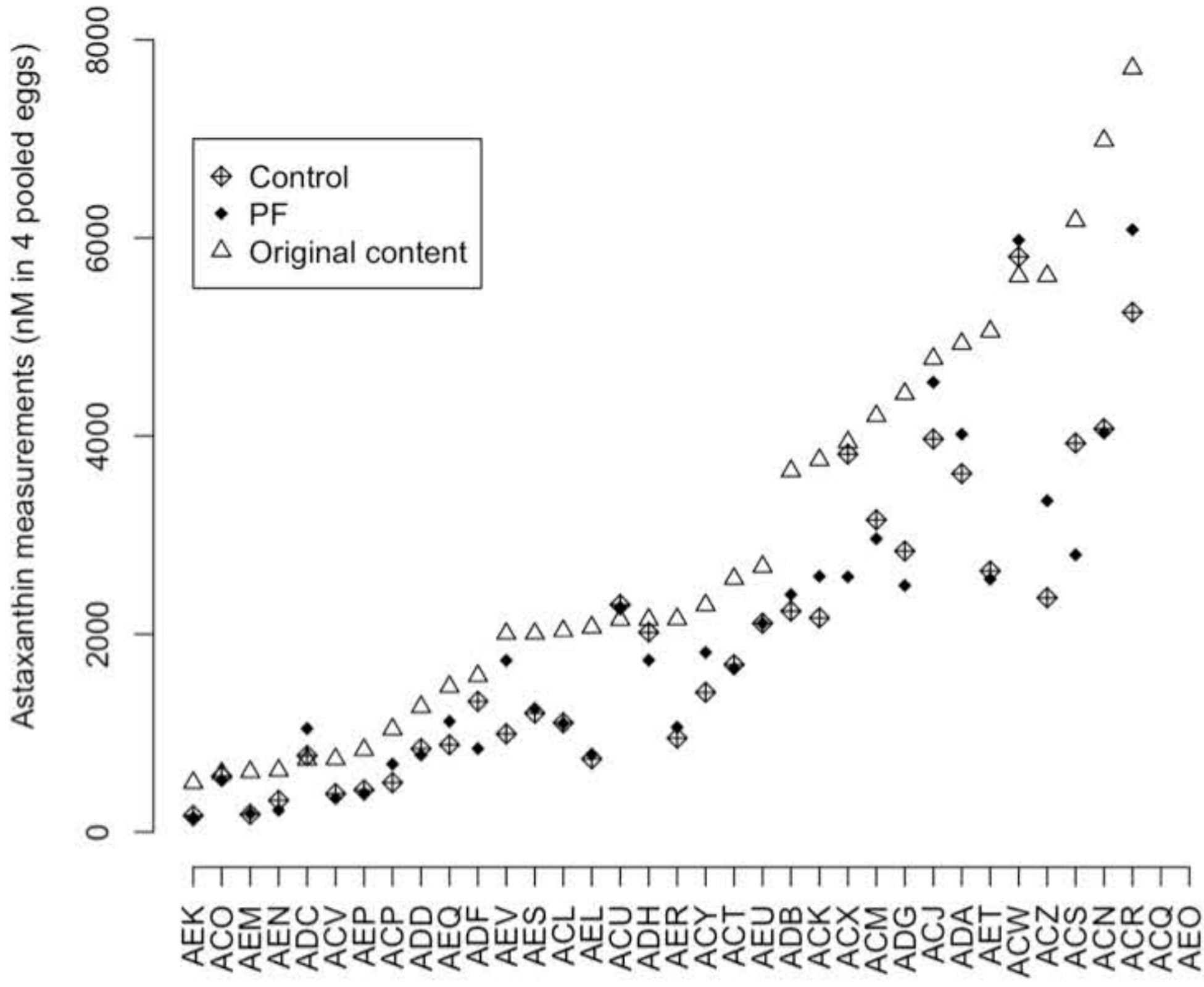

(b)

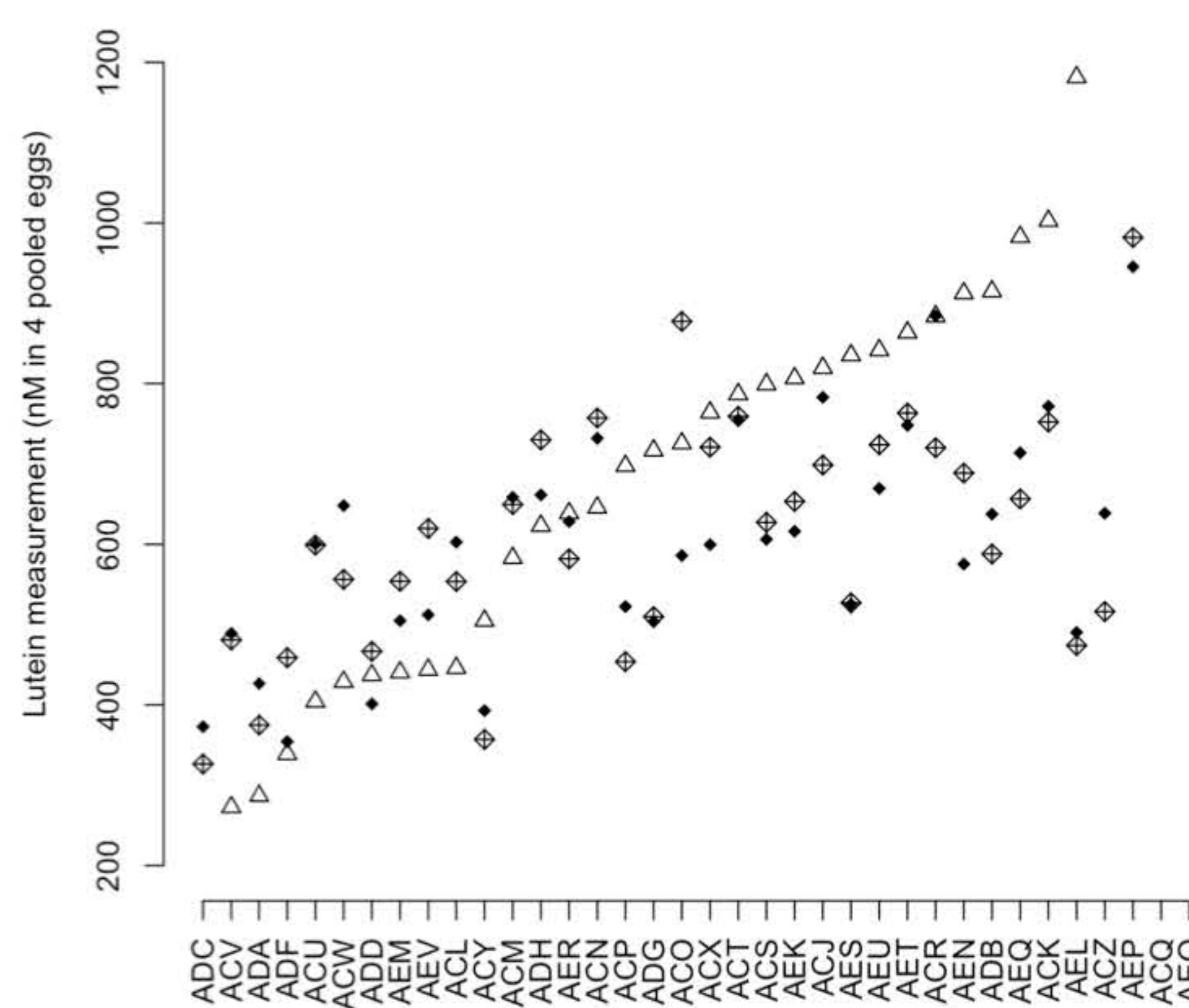

(c)

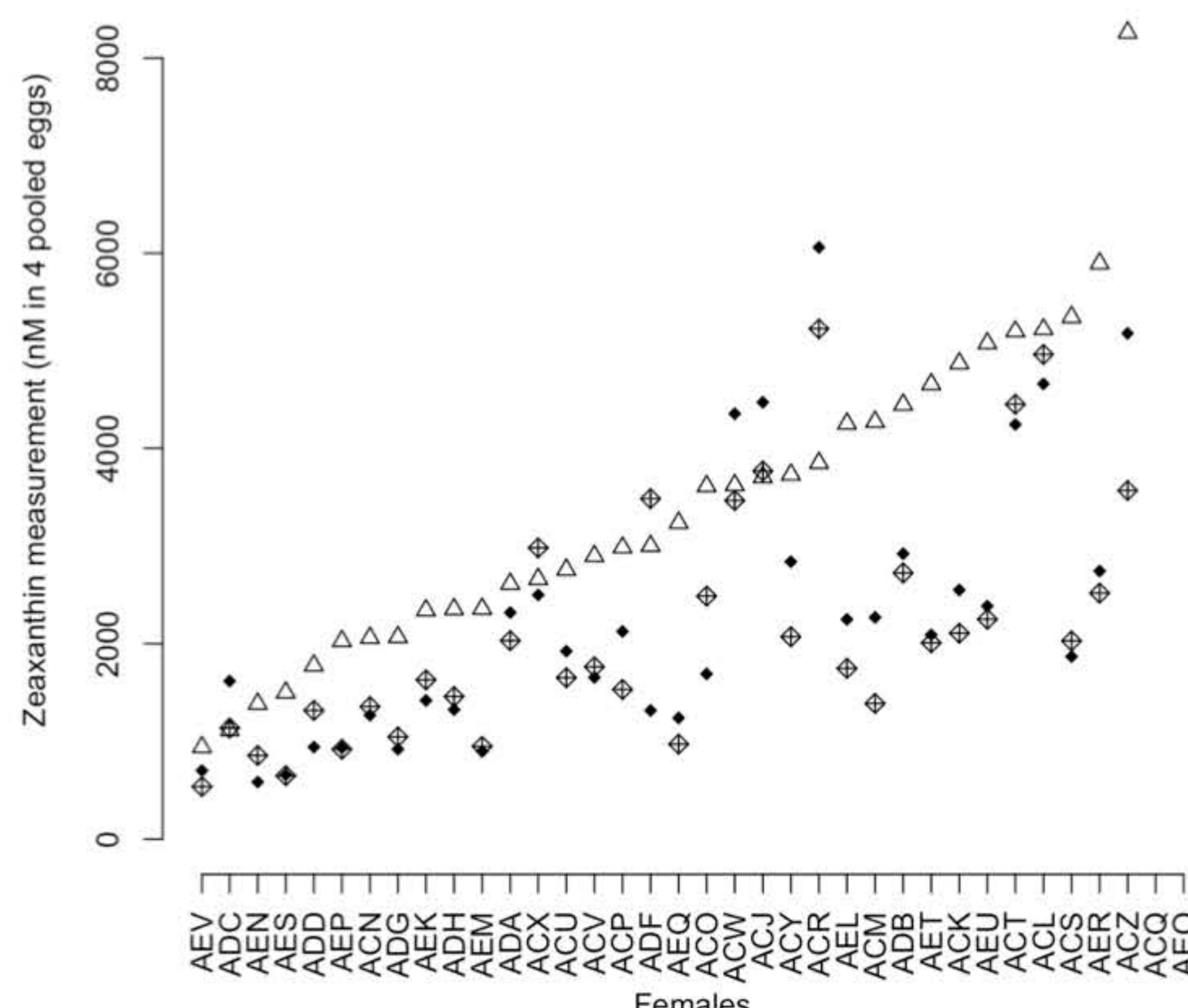

Supplement: S2 Fig — Carotenoids were quantified in eggs before fertilization (triangles), at the late-eyed development stage in the control treatment (crossed diamonds) and in the PF treatment (filled diamonds) for (a) astaxanthin, (b) lutein and (c) zeaxanthin. Data on the eggs come from Wilkins et al. [24]. (PDF) [file pone.0198834.s003.pdf]

(a)

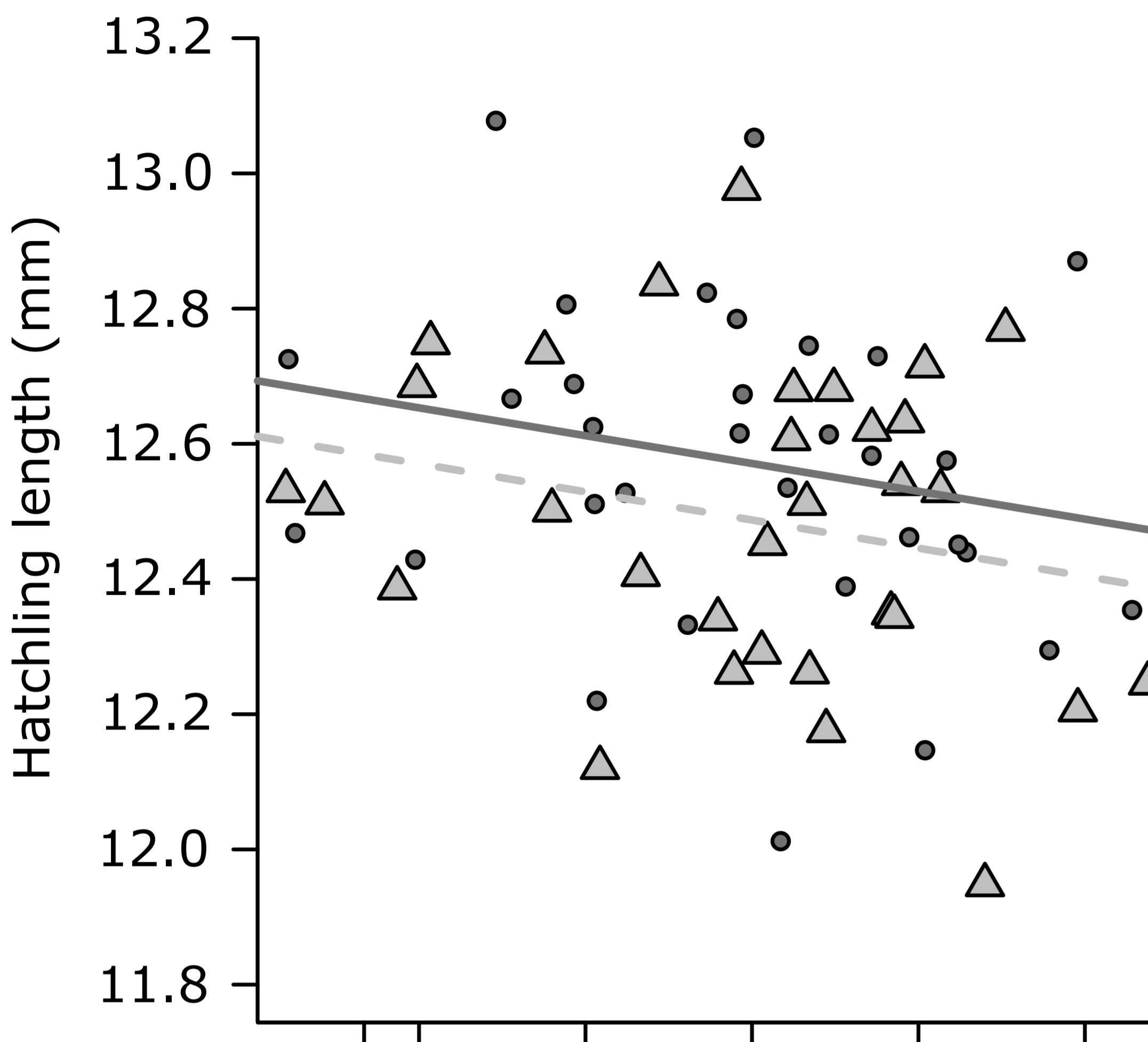

(b)

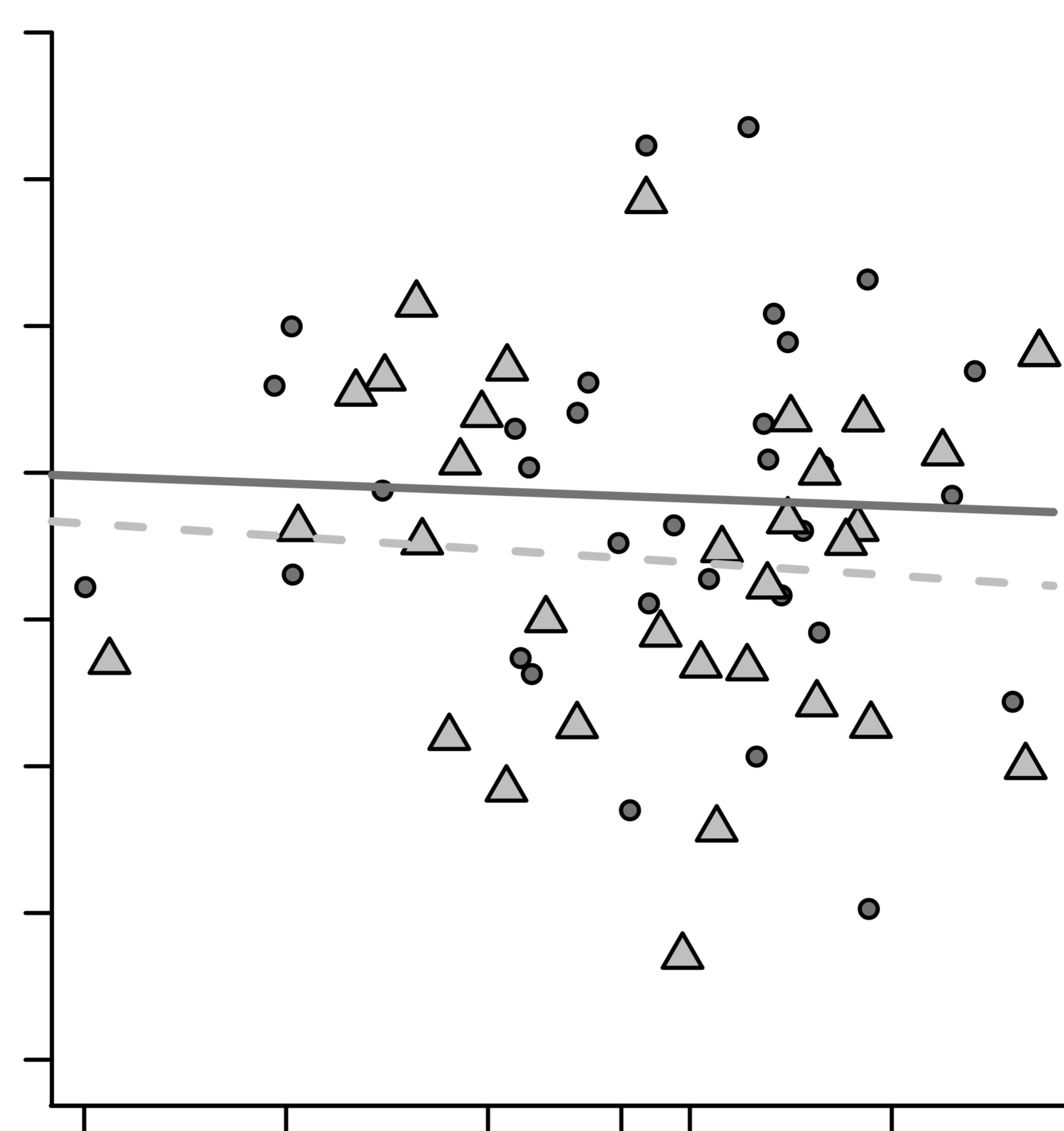

(c)

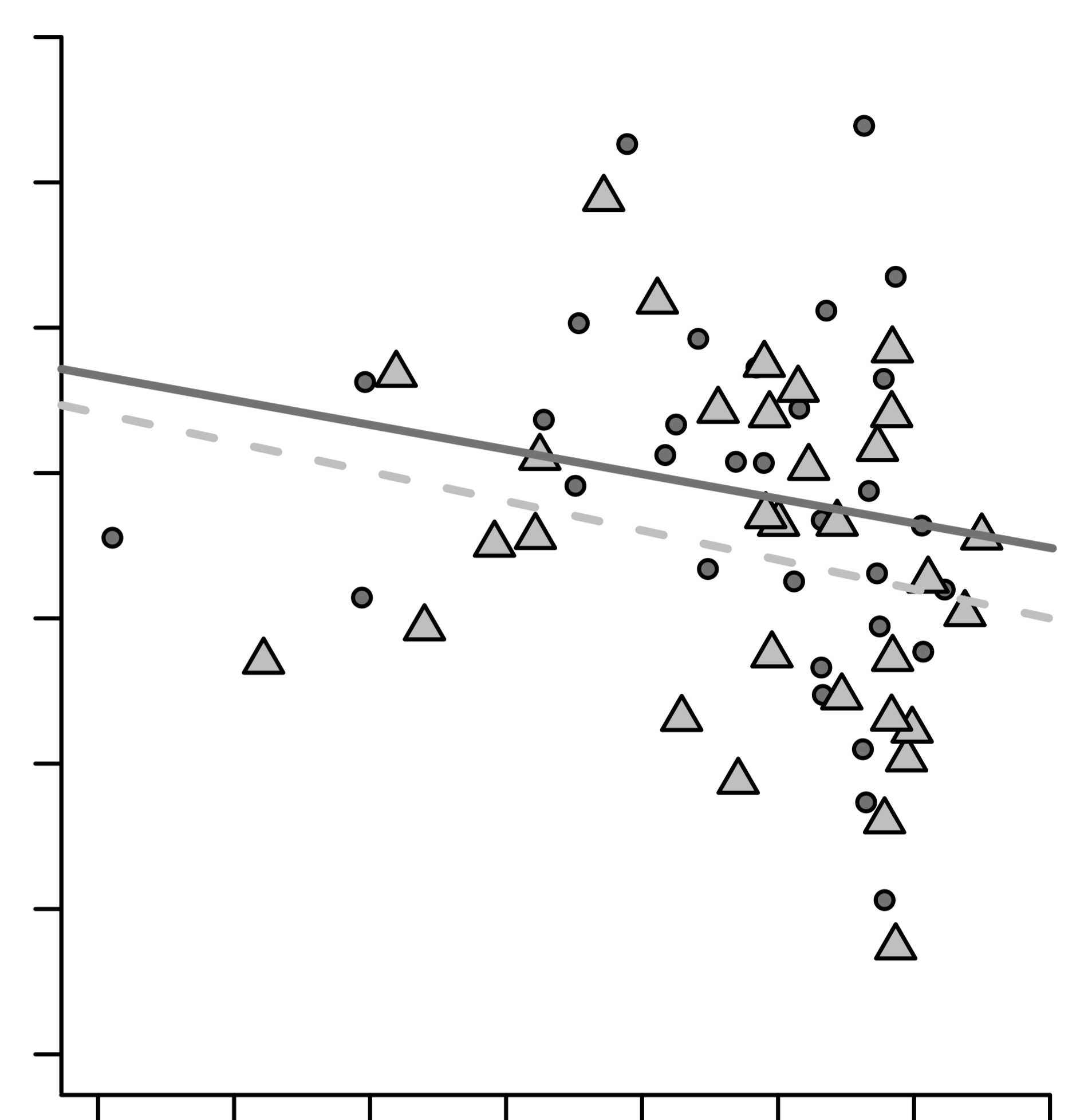

(d)

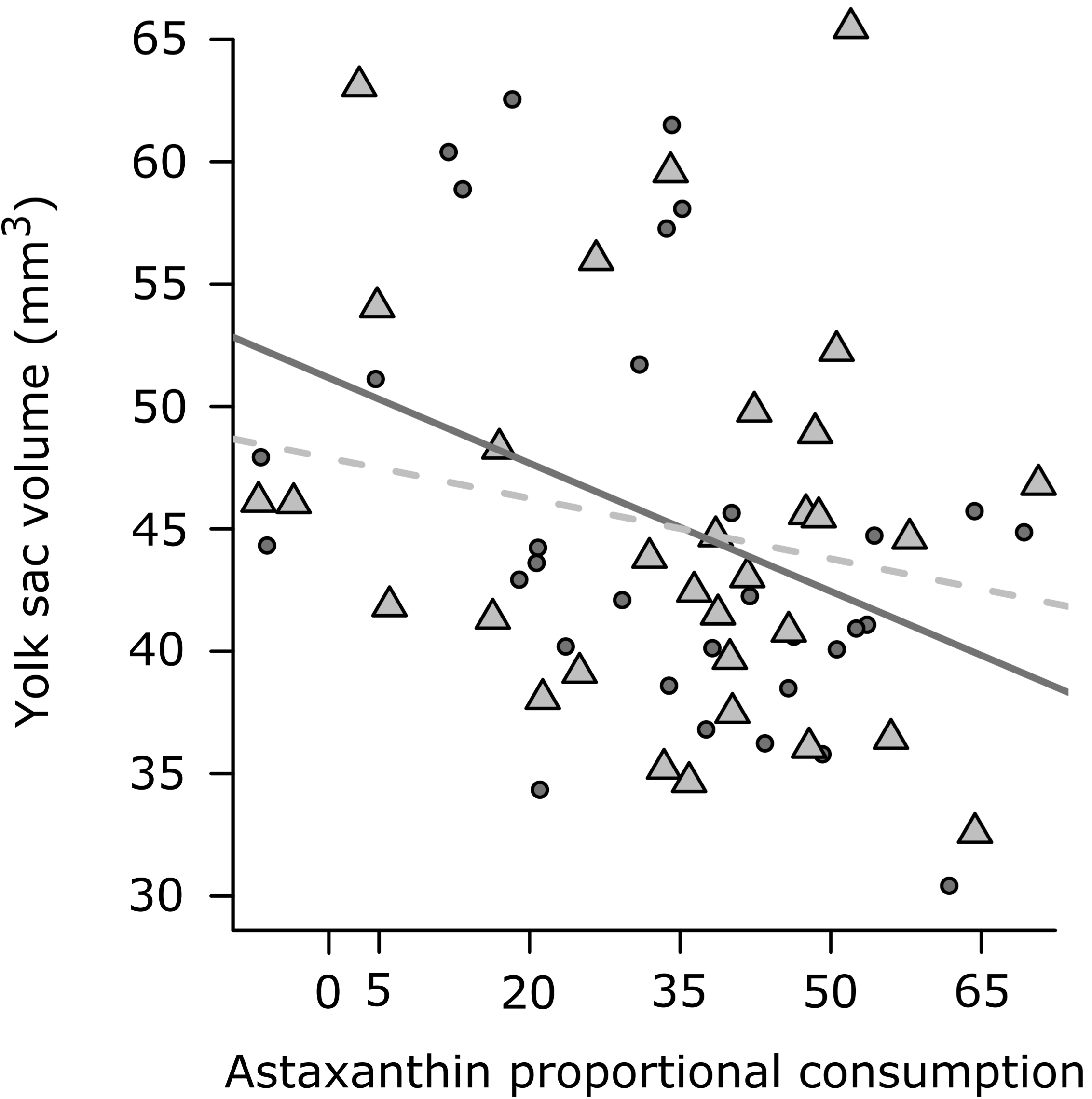

(e)

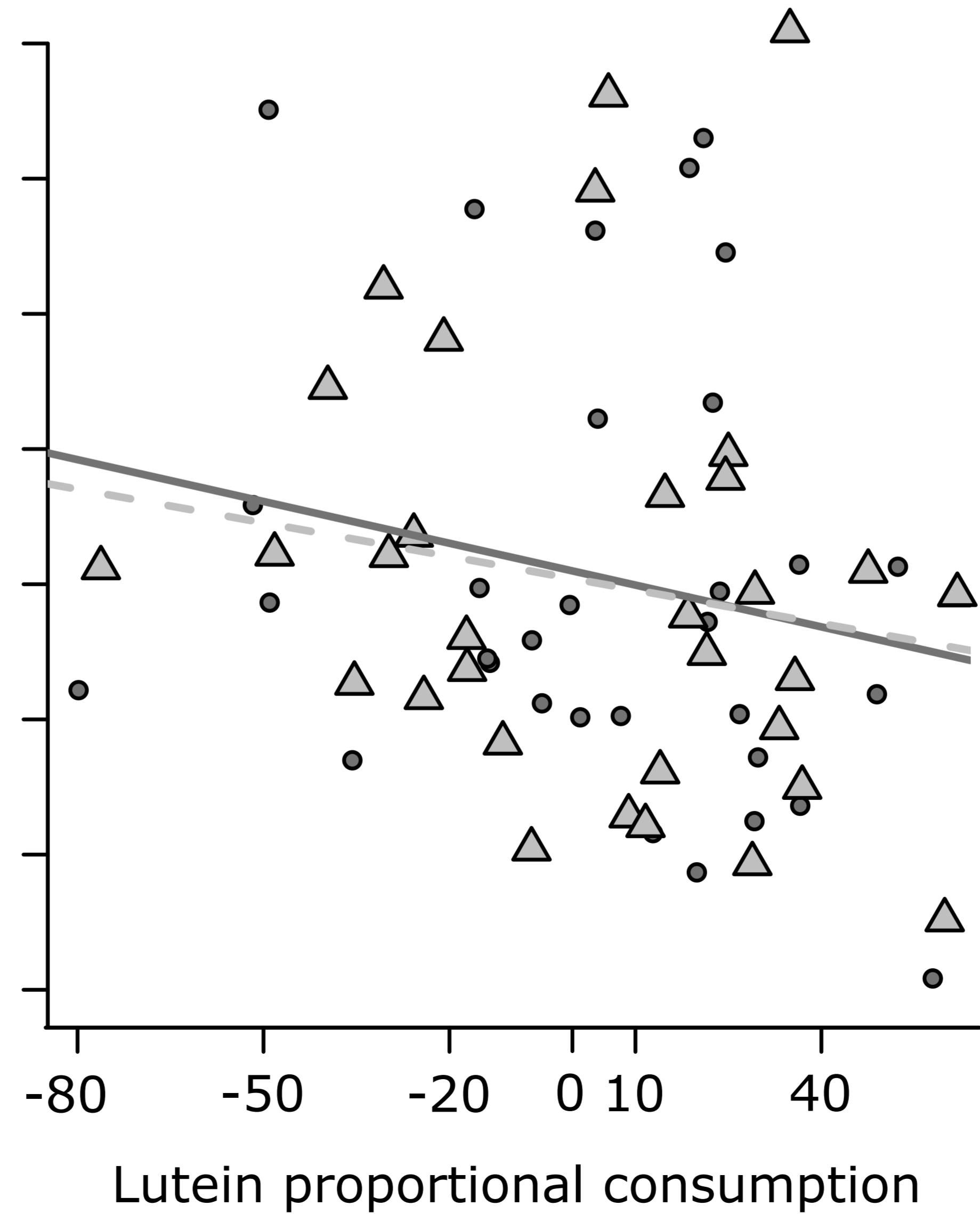

(f)

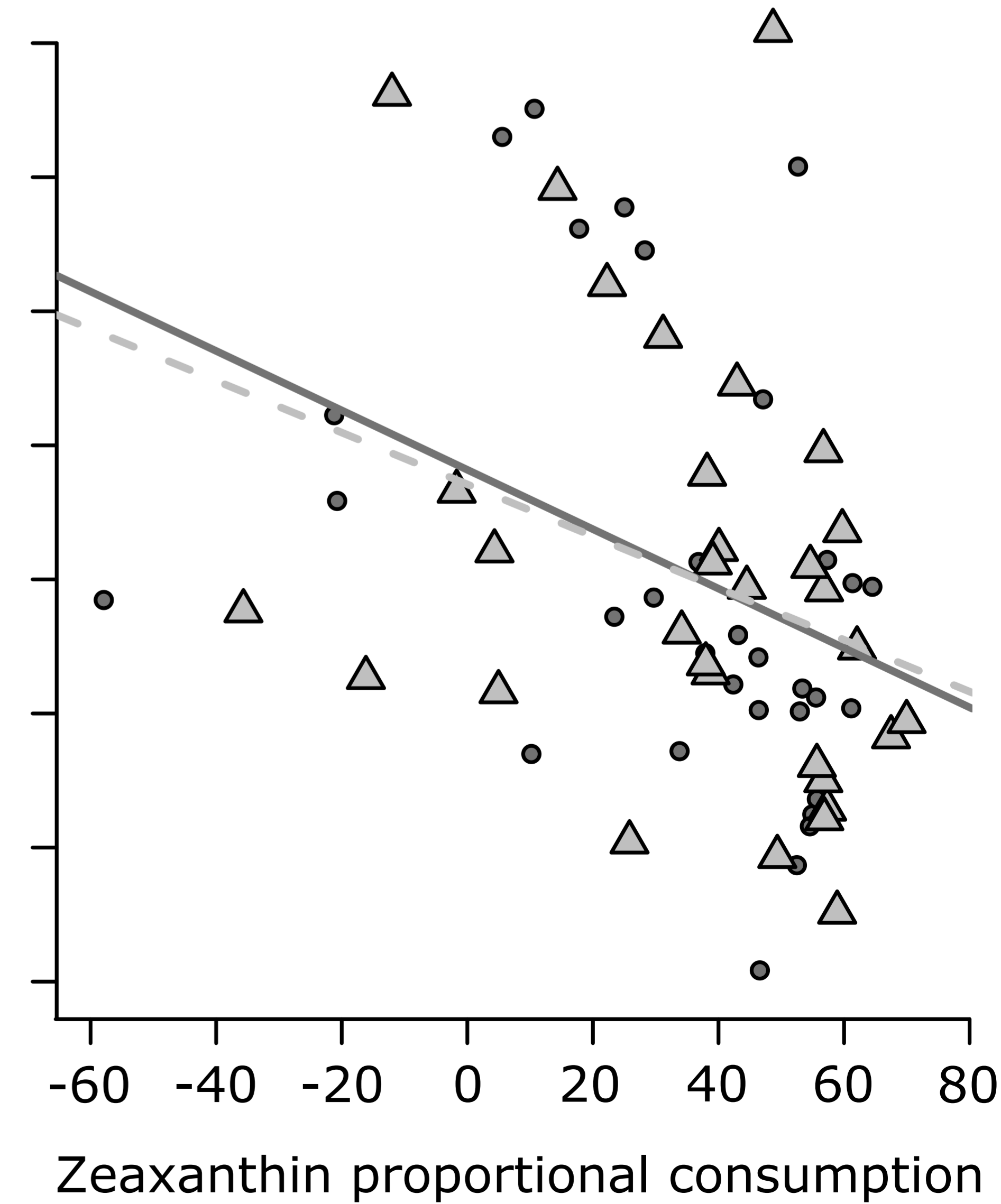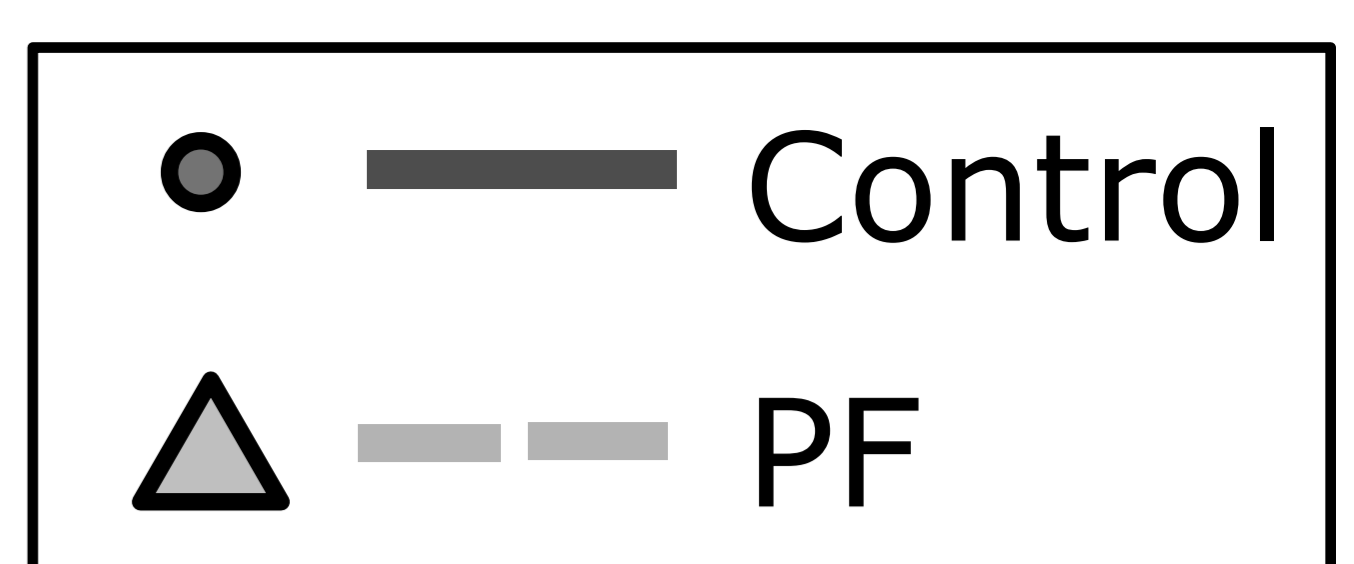

Supplement: S3 Fig — Hatchling length (a–c) and yolk sac volume at hatching (d–f) are shown for change in astaxanthin, lutein, and zeaxanthin. Changes in carotenoid contents are given for sham-treated controls (circles and solid lines) and PF treated samples (triangles and dashed lines). See Table 3 for statistics. (PDF) [file pone.0198834.s004.pdf]

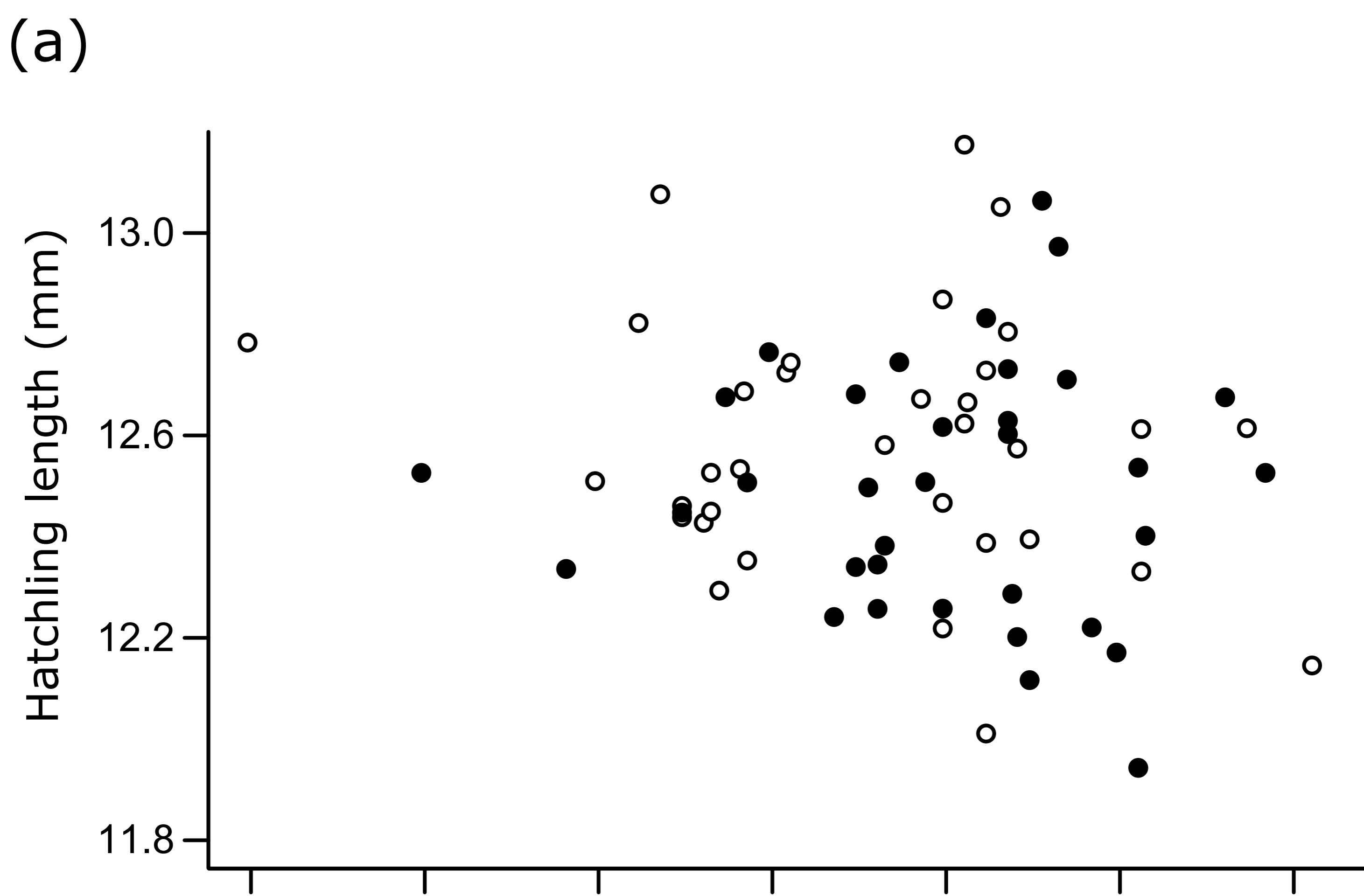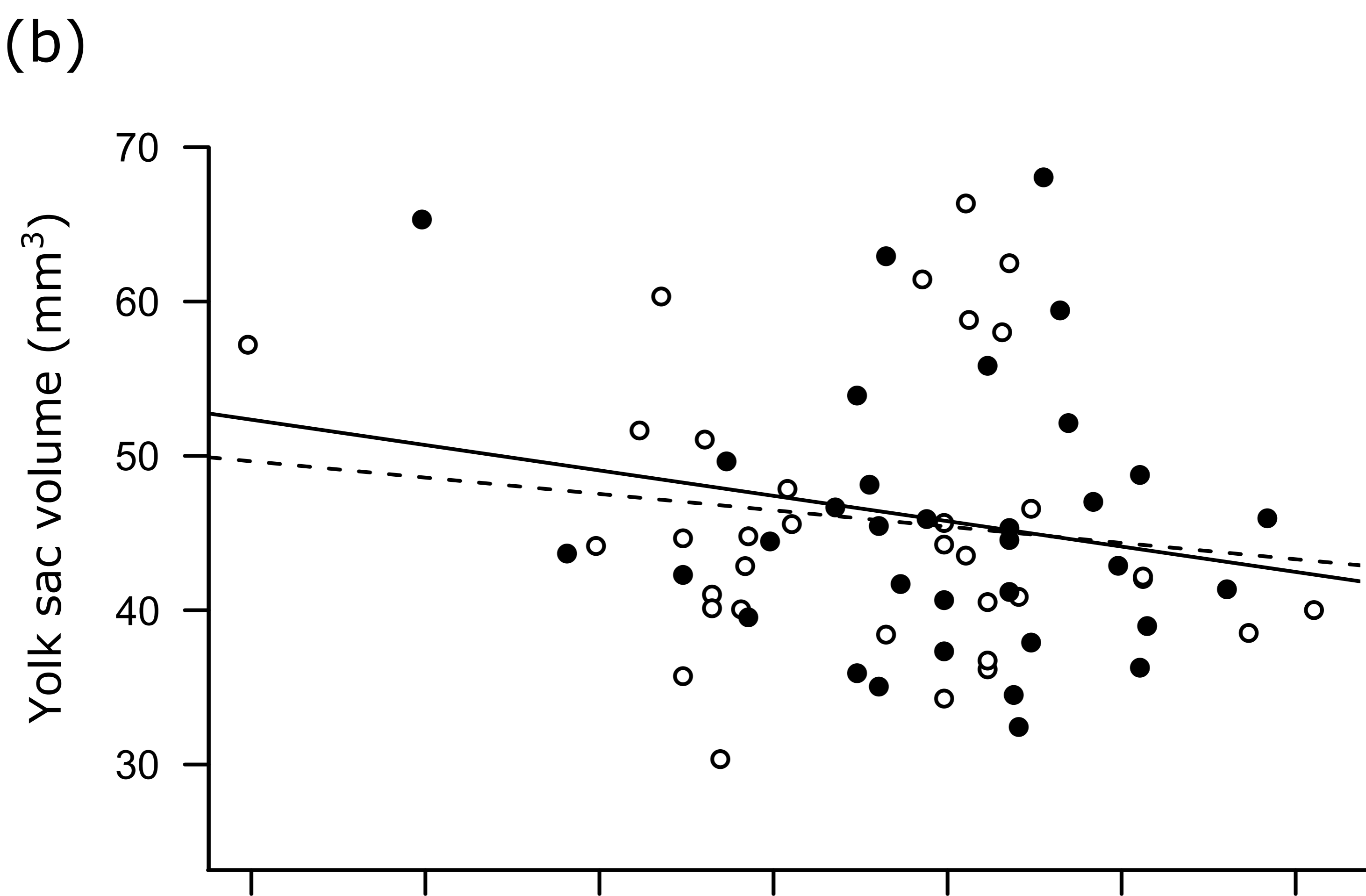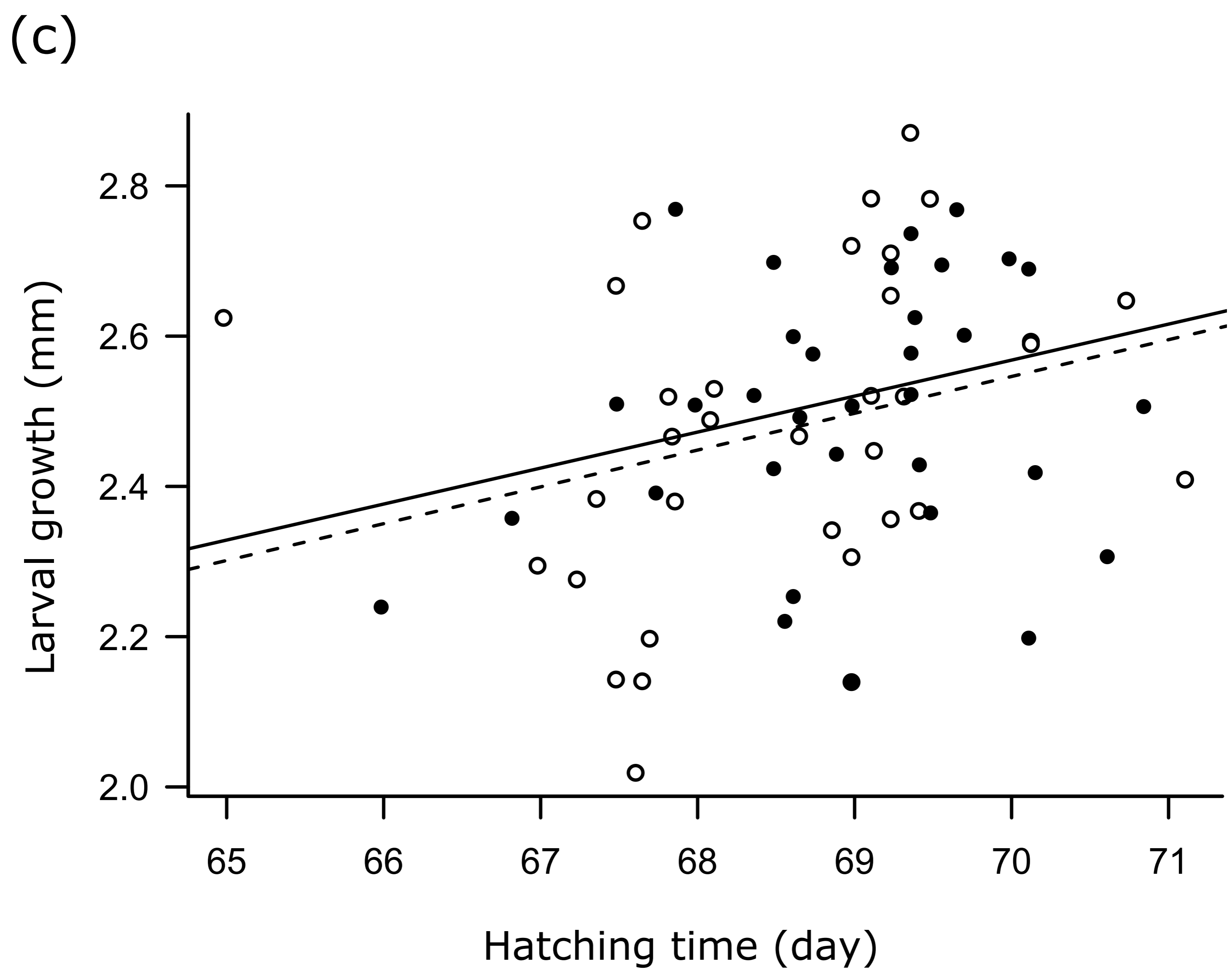

Supplement: S4 Fig — Means per families are shown for the control group (white circles, dotted lines) vs. the PF treatment (black circles, solid lines) for hatching time vs. (a) hatchling length, (b) yolk sac volume at hatching, and (c) larval growth. See Table 3 for statistics. (PDF) [file pone.0198834.s005.pdf]

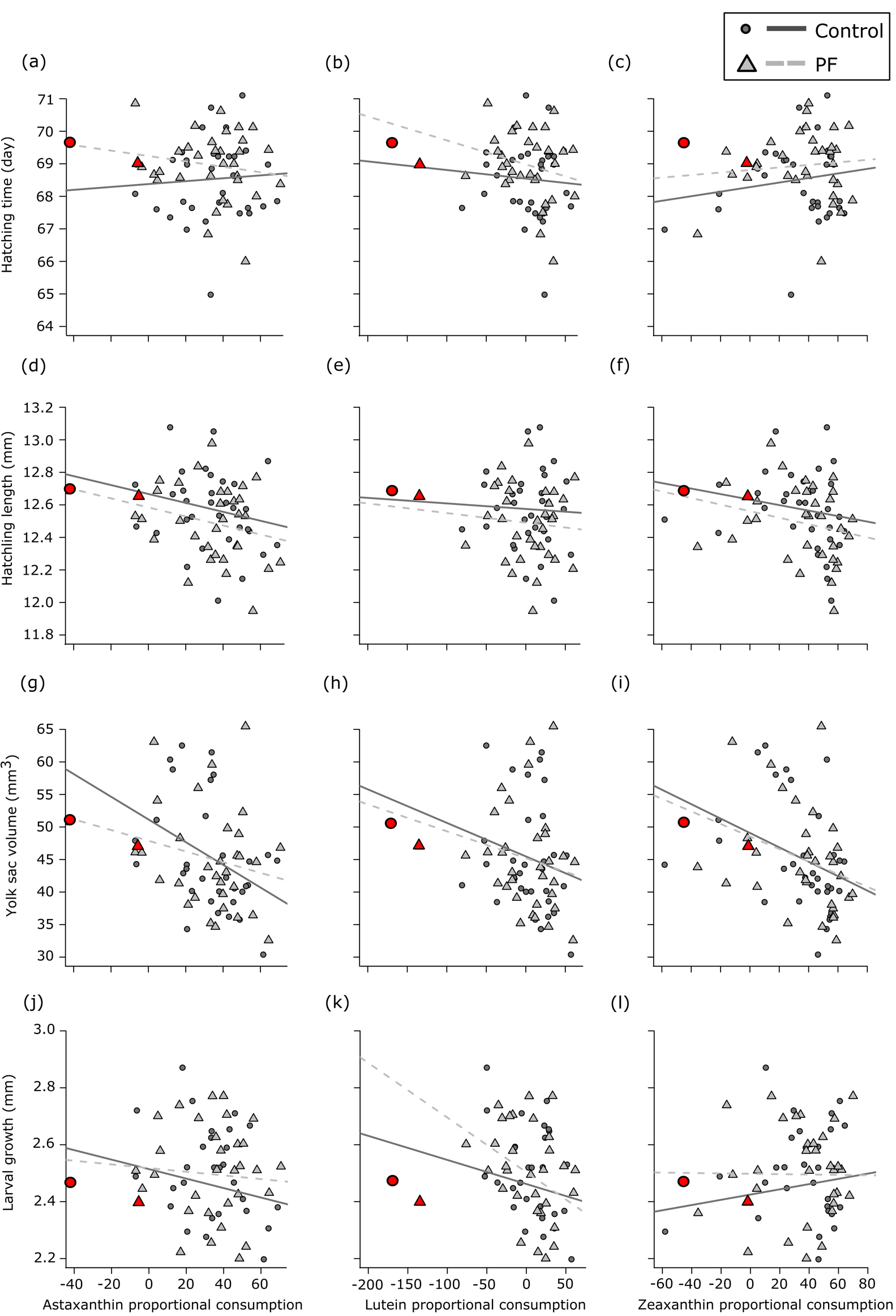

Supplement: S5 Fig — Embryo hatching time (a–c), hatchling length (d–f), yolk sac volume at hatching (g–i), and larval growth (j–l) are shown for change in astaxanthin, lutein, and zeaxanthin. Changes in carotenoid contents are given for sham-treated controls (circles and solid lines) and PF treated samples (triangles and dashed lines). Data points of the female excluded from the analyses presented in Table 3 and illustrated in Fig 3 and S3 are highlighted in red. Slopes correspond to the statistics in Table 3. (PDF) [file pone.0198834.s006.pdf]
